# Comparative genomic analysis revealed rapid differentiation in the pathogenicity-related gene repertoires between *Pyricularia oryzae* and *Pyricularia penniseti* isolated from a *Pennisetum* grass

**DOI:** 10.1186/s12864-018-5222-8

**Published:** 2018-12-13

**Authors:** Huakun Zheng, Zhenhui Zhong, Mingyue Shi, Limei Zhang, Lianyu Lin, Yonghe Hong, Tian Fang, Yangyan Zhu, Jiayuan Guo, Limin Zhang, Jie Fang, Hui Lin, Justice Norvienyeku, Xiaofeng Chen, Guodong Lu, Hongli Hu, Zonghua Wang

**Affiliations:** 1 National Engineering Research Center of JUNCAO Technology, College of Life Science, Fujian Agriculture and Forestry University, Fuzhou, 350002 China; 2 State Key Laboratory of Ecological Pest Control for Fujian and Taiwan Crops, College of Plant Protection, Fujian Agriculture and Forestry University, Fuzhou, 350002 China; 3 College of Plant Protection, Fujian Agriculture and Forestry University, Fuzhou, 350002 China; 4 College of life science, Fujian Agriculture and Forestry University, Fuzhou, 350002 China; 5 Institute of Oceanography, Minjiang University, Fuzhou, 350108 China

**Keywords:** *Pennisetum*, *Pyricularia*, PacBio sequencing, Pathogenesis-related genes, Comparative genomic analysis

## Abstract

**Background:**

A number of *Pyricularia* species are known to infect different grass species. In the case of *Pyricularia oryzae* (syn. *Magnaporthe oryzae*), distinct populations are known to be adapted to a wide variety of grass hosts, including rice, wheat and many other grasses. The genome sizes of *Pyricularia* species are typical for filamentous ascomycete fungi [~ 40 Mbp for *P. oryzae*, and ~ 45 Mbp for *P. grisea*]. Genome plasticity, mediated in part by deletions promoted by recombination between repetitive elements [Genome Res 26:1091-1100, 2016, Nat Rev Microbiol 10:417-430,2012] and transposable elements [Annu Rev Phytopathol 55:483-503,2017] contributes to host adaptation. Therefore, comparisons of genome structure of individual species will provide insight into the evolution of host specificity. However, except for the *P. oryzae* subgroup, little is known about the gene content or genome organization of other *Pyricularia* species, such as those infecting *Pennisetum* grasses.

**Results:**

Here, we report the genome sequence of *P. penniseti* strain P1609 isolated from a *Pennisetum* grass (JUJUNCAO) using PacBio SMRT sequencing technology. Phylogenomic analysis of 28 Magnaporthales species and 5 non-Magnaporthales species indicated that P1609 belongs to a *Pyricularia* subclade, which is genetically distant from *P. oryzae*. Comparative genomic analysis revealed that the pathogenicity-related gene repertoires had diverged between P1609 and the *P. oryzae* strain 70–15, including the known avirulence genes, other putative secreted proteins, as well as some other predicted *Pathogen-Host Interaction* (*PHI*) genes. Genomic sequence comparison also identified many genomic rearrangements relative to *P. oryzae*.

**Conclusion:**

Our results suggested that the genomic sequence of the *P. penniseti* P1609 could be a useful resource for the genetic study of the *Pennisetum*-infecting *Pyricularia* species and provide new insight into evolution of pathogen genomes during host adaptation.

**Electronic supplementary material:**

The online version of this article (10.1186/s12864-018-5222-8) contains supplementary material, which is available to authorized users.

## Background

*Pyricularia* was established by Saccardo to accommodate a type of fungal species based on pyriform conidia when the first species of this pathogen, *Pyricularia grisea*, was isolated from crabgrass (*Digitaria sanguinalis* L.) [1]. *Pyricularia* became an important research focus due to rice blast disease and now wheat blast caused by *Pyricularia oryzae* (syn. *Magnaporthe oryzae*) [2–4]. To date, 100 plant genera comprising 256 species have been documented as hosts of *Pyricularia* species (https://nt.ars-grin.gov/fungaldatabases/), among which 54 genera belong to the Poaceae family. Seven *Pyricularia* species (including one unidentified species) have been isolated from *Pennisetum* spp., a large genus in the Poaceae family, and more than one *Pyricularia* species can be found on the same *Pennisetum* species. For instance, 4 *Pyricularia* species, namely, *P. penniseti*, *P. penniseticola*, *P. setariae* and *Pyricularia sp*. have been found on *P. typhoides* [5, 6]*.*

The genome sequence of *P. oryzae* strain 70–15, a strain produced by an initial cross of rice-infecting isolate 104–3 and the weeping love grass isolate AR4 [7]. A series of selections from crosses, including three generations crossing to the rice isolate Guy11 generated fertile rice infecting strains that have facilitated studies of developmental and pathogenic mechanisms of the blast fungus and helped *P. oryzae* to become one of the most important fungal models of plant pathogenesis [8, 9]. Since the publication of the *P. oryzae* strain 70–15 genome, more field isolates were sequenced and assembled, including Ina168, HN9311, FJ81278, Y34, P131, 98–06 and Guy11 [10–14]. Comparative genomic analyses and functional studies of these strains revealed genome plasticity and the involvement of the lineage specific genes in pathogenicity [12, 14]. More recently, facilitated by the fast development of sequencing technologies, a number of field isolates from rice, as well as isolates from different grass and cereal hosts, were sequenced and subjected to population-level analyses, revealing host immunity as a major force driving specialization [2, 15–18]. However, genomes of most of the species of the *Pyricularia* complex remain unexplored. For example, among the 7 identified *Pyricularia* species isolated from *Pennisetum* grasses [5], only *P. pennisetigena* was recently sequenced [2].

Here, we reported the whole-genome sequence of *P. penniseti* [19] isolated from a *Pennisetum* grass JUJUNCAO (*Pennisetum giganteum* Z. X. Lin). JUJUNCAO was originally developed as a biomass crop for the cultivation of edible mushrooms by Lin et al, and later became a versatile grass that is used as forage for cattle and sheep, material for biofuel production, and a tool for the prevention of soil erosion [20–22]. We have recently isolated a fungus, producing pyriform-shaped conidia from leaf spots of JUJUNCAO. One isolate, P1609, caused a typical blast fungal disease symptom on JUJUNCAO, showing small, round or elliptical lesions as an initial symptom with spindle shaped, grayish to tan necrotic lesion centers, and yellow halos at a later disease stage. The morphologic and phylogenetic analyses distinguished P1609 from other identified *Pyricularia* species, but it was not clearly distinguishable from the *P. penniseti* reported in 1970 in India [5, 23]. We therefore identified this fungus as *P. penniseti* [19]*.* In this study, we performed genome sequencing of this strain, aiming for a proper classification of this fungus in the *Pyricularia* population and identification of genes that may be involved in the adaptation of this fungus to JUJUNCAO.

## Results

### Genome sequencing and assembly

**Fig. 1:**
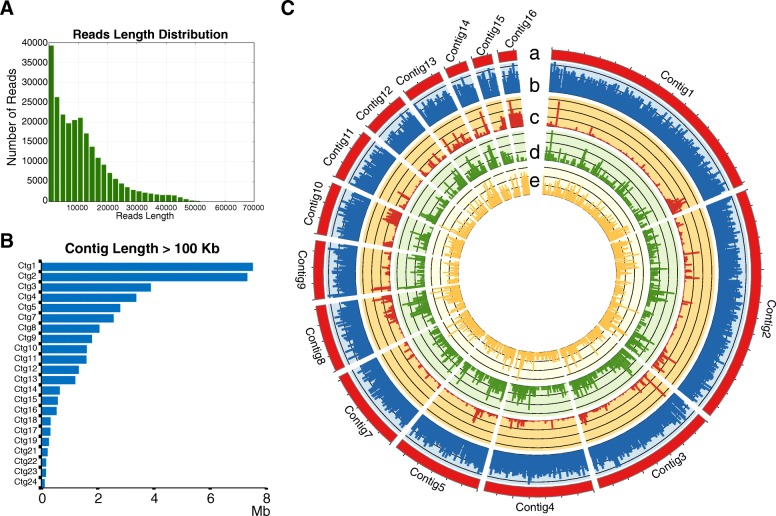
PacBio sequencing and genome assembly of P1609. **a** Reads length distribution. **b** Contig length of assembled contig > 100 Kb. **c** Overview of P1609 genome. (Track a) Contig1 to contig16 of P1609, (track b) gene density, (track c) Transposon elements density, (track d) secreted proteins density, (track e) unique gene (compared with *P. oryzae*, *N. crassa*, *F. graminearum* and *C. gloeosporioides*) density of P1609 per 50 Kb

We sequenced the P1609 genome with the long-read PacBio technology. In total, 312,061 reads with 8.6 Kb average lengths were obtained, representing about 60-fold coverage of the genome (Fig. 1a). The genome sequence was assembled with the HAGP pipeline [24], resulting in a total assembly space of 41.82 Mb (Table 1), similar to assemblies of other isolates sequenced by PacBio [10]. The assembly contains 53 contigs, with the N50 of 3.4 Mb and the largest contig of 7.56 Mb (Fig. 1b). Contigs > 1 Mb cover 89.7% and contigs > 100 Kb cover 98.5% of the genome (Fig. 1b; Table 1), indicating long-continuity of the assembly. The GC content of the assembly is 50.3%, similar to genomes of *Pyricularia* isolates from different host plants, which range from 48.6 to 51% [18]. Genome annotation identified 13,102 genes with average gene size of 1758 bp, fairly evenly dispersed on contigs (Table 2, Fig. 1c, track b).

**Table 1:** Details of sequencing reads and genome assembly of P1609

Feature	Value
Number of Subreads	312,061
Total Length of Subreads (bp)	2,688,966,115
Mean Coverage	59
Polished Contigs	53
Maximum Contig Length (bp)	7,555,856
N50 Contig Length (bp)	3,411,241
Sum of Contig Lengths (Mb)	41.82
GC level	50.30%
length > 1 Mb	89.70%
length > 100 Kb	98.50%

**Table 2:** Details of genome annotation of P1609

Category	Value
Total TE	7.67%
LINEs	1.50%
LTR elements	4.27%
DNA elements	0.63%
Unclassified	1.27%
Gene Number	13,102
Average Gene Length (bp)	1758
Secreted Protein Number	1409

De novo repeat sequence analysis identified 7.67% repeat sequences, among which 4.27% were Gypsy and Copia, the two long terminal repeats (LTR)-type retrotransposons. Although the overall content of repeat sequences in P1609 is less than that of the sequenced *P. oryzae* strains [8, 10, 12], the high proportion of Gypsy and Copia in repeat sequence is similar to that of the reported *P. oryzae* strains [25]. The TEs are not evenly distributed along the contigs, with some contigs being highly enriched with TEs (Fig. 1c, track c), suggesting that the P1609 genome also underwent transposon expansion as observed in other plant pathogens [26].

### Comparative and phylogenetic analysis

**Fig. 2:**
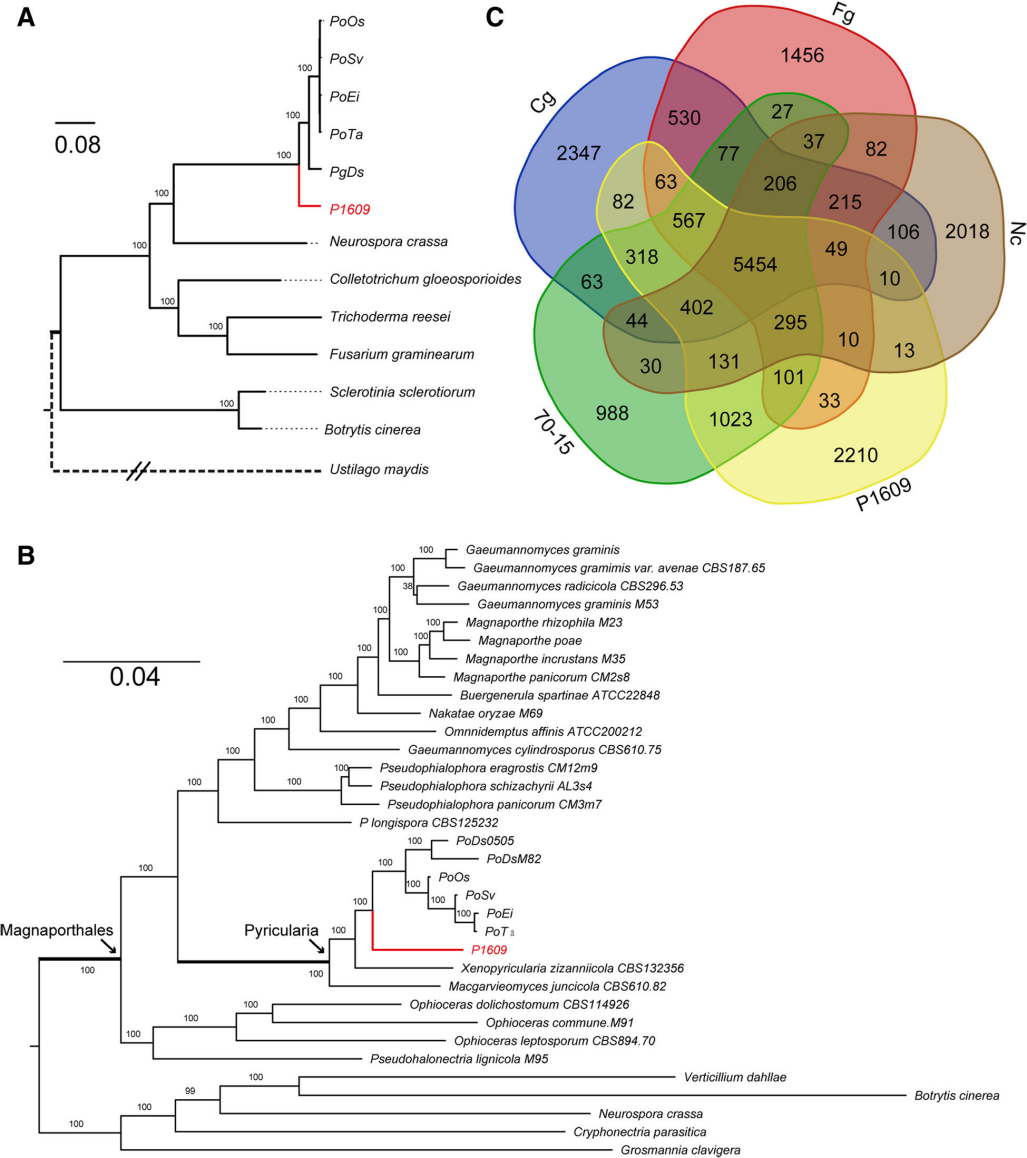
Phylogenetic and comparative genomic study of P1609. **a** Phylogenomic tree of P1609 with *Botrytis cinereal*, *Colletotrichum gloeosporioides*, *Fusarium graminearum*, *Neurospora crassa*, *Sclerotinia sclerotiorum*, *Trichoderma reesei* and *Ustilago maydis* as well as *Pyricularia* isolates collected from *O. sativa* (70–15), *T. aestivum* (PoTa), *D. sanguinalis* (PgDs), *S. viridis* (PoSv), and *E. indica* (PoEi) based on 2051 single copy genes. The values of all of the branches are 100. **b** Maximum likelihood tree of P1609 and 28 Magnaporthales species, as well as 5 Sordariomycetes used as outgroup species based on 82,715 amino acid positions derived from 226 genes. **c** Venn diagram showed an overlap of gene families among P1609, *Pyricularia* rice isolates (PoOs), *C. gloeosporioides* (Cg), *F. graminearum* (Fg) and *N. crassa* (Nc)

To understand the genetic relationship of P1609 with other fungal phytopathogens, we generated a phylogenetic tree of P1609 with *Botrytis cinerea*, *Colletotrichum gloeosporioides*, *Fusarium graminearum*, *Neurospora crassa*, *Pyricularia grisea* (Pg), *Pyricularia oryzae* (Po), *Sclerotinia sclerotiorum*, *Trichoderma reesei* and *Ustilago maydis*. Since P1609 showed a close morphological relationship with *P. oryzae* isolates, we included *Pyricularia* isolates collected from different host plants, including *Oryza sativa* (PoOs), *Triticum aestivum* (PoTa), *Digitaria sanguinalis* (PgDs), *Setaria viridis* (PoSv), and *Eleusine indica* (MoEi). In total, 2051 single-copy genes shared by all the examined genomes were selected to infer phylogeny [27]. The resulting phylogenetic tree indicated that P1609 is more distantly related to PoOs, PoTa, PoSv, and PoEi (*P. oryzae*) than PgDs (*P. grisea*) (Fig. 2a). We then estimated divergence time of P1609 and *Pyricularia* isolates by assuming a constant molecular clock calibrated in a previous study [28, 29], which estimated the divergence of *Neurospora* and *Pyricularia* at about 200 million years ago (MYA). The estimation indicated that P1609 and *Pyricularia* isolates diverged at about 31 MYA, earlier than the divergent time of rice- and *S. viridis*-infecting isolates (about 10,000 years ago) [28, 30]. The phylogenetic tree also indicated that P1609 is a member of Magnaporthales, but is distantly related to the *P. oryzae*. To further determine exactly where P1609 localized in Magnaporthales, we also generated a phylogenomic tree of P1609 with 28 Magnaporthales species and 5 non-Magnaporthales species using amino acid sequences of 226 conserved orthologous genes as described [31]. The result showed that P1609 was localized in the *Pyricularia* subclade, and was closer to *P. oryzae* than *Xenopyricularia zizaniicola* (Fig. 2b), indicating that P1609 is a *Pyricularia* species.

We next conducted a comparative genomic study of P1609 with 70–15 (the reference isolate of *P. oryzae*), *N. crassa* (Nc), *F. graminearum* (Fg) and *C. gloeosporioides* (Cg) using OrthoFinder [27]. The five organisms share 5454 of gene families with each other, which covers 50.2% of the gene set of P1609 (Fig. 2c). Notably, the number of P1609 specific genes in this comparison was 2210. This is about twice the number of 70–15 specific genes. Although most of these P1609 specific genes had no functional annotations, Pfam annotation indicated that some encode carbohydrate-active enzymes (CAZymes) involved in polysaccharide metabolism pathways (Additional file 1: Figure S1; Additional file 2: Table S1).

To identify genes under positive selection, Ka/Ks ratios of the orthologous genes in P1609 versus 70–15 were calculated, with the assumption that Ka/Ks ratio > 1 suggested a positive selection of the gene [11]. Among the 5991 pairs of orthologs of P1609 and 70–15 scanned, 6 genes with Ka/Ks > 1 were identified in P1609 (Table 3), suggestive of positive selection on these genes in P1609 during the adaptation to JUJUNCAO. These genes are involved in different secondary metabolic pathways (See Discussion).

**Table 3:** Positively selected genes in P1609

Protein ID	Ka	Ks	Ka/Ks	*P*-Value (Fisher)	Description
P1609_7184	0.707979	0.440748	1.60631	0.00697146	Unknown
P1609_5032	0.550905	0.343609	1.60329	1.98443E-06	Isoamyl alcohol oxidase
P1609_3360	0.183224	0.118517	1.54597	0.00293936	Glycerol uptake protein 1
P1609_791	0.302231	0.199786	1.51277	9.75093E-06	Folylpolyglutamate synthase
P1609_7869	0.491873	0.328109	1.49911	0.0296442	Thioredoxin reductase
P1609_1006	0.40206	0.275456	1.45962	0.0288585	Spore surface glycoprotein BclB

### Gene categories involved in pathogenicity

Plant pathogenic fungi employed diverse gene repertoires to invade host plants and subvert host immune systems, which include effectors, carbohydrate-active enzymes (CAZymes), other secreted enzymes and fungal secondary metabolisms [32, 33]. To understand the differences between *P. penniseti* and *P. oryzae* secretomes, we examined the 1409 putative secreted proteins of P1609 predicted by SignalP [34], and found 236 were unique in P1609 (Additional file 3: Table S2). By contrast, 165 putative effectors identified in the genome of 70–15, the rice blast reference fungal genome, were absent in the P1609 genome (Additional file 4: Table S3). Notably, all known avirulence genes (*AVRs*) from the rice-infecting isolate genomes were absent in P1609 genome.

Given the important roles of CAZymes in enabling plant pathogens to break down the plant cell wall [35], we next compared CAZymes between P1609 and 70–15. Our BLASTp search results showed that P1609 contains more predicted CAZyme-coding genes than 70–15 (Additional file 2: Table S1). Detailed analysis showed that the P1609 genome encodes five unique CAZymes belonging to five families, namely CBM61, GH117, GH35, GH65 and PL24, respectively (Additional file 2: Table S1). While it has six copies of GH28 pectinases (three copies in *P. oryzae and P. grisea*; Additional file 5: Figure S2). The high representation of carbohydrate-active enzymes may be related to the adaptation to the host *P. giganteum*. We then further analyzed the distribution of annotated PHI genes in P1609. In total, we identified 1692 potential PHI genes belonging to 1154 gene families (Additional file 6: Table S4). Interestingly, we found that several PHI genes exhibited great expansion in P1609 genome. For instance, *MGG_12656*, a gene involved in virulence in *P. oryzae*, has 107 homologs in P1609, and ChLae1 contributing to toxin production and virulence in the maize pathogen *Cochliobolus heterostrophus* has 17 homologs in P1609 [36, 37]. Compared with 70–15, 35 PHI genes were unique in P1609 (Table 4), most of which had highly similar homologs in *Fusarium* (*Gibberella*).

**Table 4:** Unique Pathogen Host Interaction (PHI) genes in P1609

Protein ID	PHI ID	Reference Organism	Phenotypes
P1609_2497_494aa	PHI:115	*Cochliobolus carbonum*	Unaffected pathogenicity
P1609_11506_262aa, P1609_12781_266aa, P1609_4501_233aa, P1609_683_218aa	PHI:698	*Vibrio cholerae*	Reduced virulence
P1609_11592_161aa, P1609_4614_892aa	PHI:1284	*Fusarium graminearum*	Unaffected pathogenicity
P1609_11335_610aa	PHI:1803	*Fusarium graminearum*	Unaffected pathogenicity
P1609_9374_326aa	PHI:2394	*Fusarium graminearum*	Increased virulence
P1609_1007_600aa	PHI:225	*Fusarium solani*	Reduced virulence
P1609_13007_833aa	PHI:1714	*Fusarium graminearum*	Lethal
P1609_3609_435aa, P1609_5044_426aa, P1609_8181_460aa, P1609_960_144aa	PHI:1455	*Fusarium graminearum*	Unaffected pathogenicity
P1609_11548_383aa, P1609_5843_397aa	PHI:1823	*Fusarium graminearum*	Unaffected pathogenicity
P1609_2665_64aa	PHI:1421	*Fusarium graminearum*	Lethal
P1609_2607_206aa, P1609_2608_820aa	PHI:3418	*Staphylococcus saprophyticus*	Mixed outcome
P1609_11380_688aa	PHI:1809	*Fusarium graminearum*	Lethal
P1609_7767_257aa	PHI:317	*Aspergillus fumigatus*	Reduced virulence
P1609_6736_447aa	PHI:323	*Verticillium fungicola*	Reduced virulence
P1609_13_169aa	PHI:1147	*Fusarium pseudograminearum*	Unaffected pathogenicity
P1609_2376_825aa	PHI:1871	*Fusarium graminearum*	Lethal
P1609_10320_326aa	PHI:1522	*Fusarium graminearum*	Lethal
P1609_11485_355aa, P1609_2130_433aa	PHI:3116	*Pseudomonas syringae*	Mixed outcome
P1609_10203_738aa	PHI:1815	*Fusarium graminearum*	Unaffected pathogenicity
P1609_11600_2486aa	PHI:2628	*Salmonella enterica*	Reduced virulence, 663
P1609_11388_1000aa	PHI:3076	*Candida parapsilosis*	Mixed outcome
P1609_11869_369aa	PHI:2375	*Alternaria brassicicola*	Mixed outcome
P1609_5035_383aa	PHI:233	*Cochliobolus carbonum*	Reduced virulence
P1609_5702_488aa	PHI:1271	*Fusarium graminearum*	Unaffected pathogenicity
P1609_11864_596aa	PHI:2380	*Alternaria brassicicola*	Mixed outcome

### Chromosome rearrangements

**Fig. 3:**
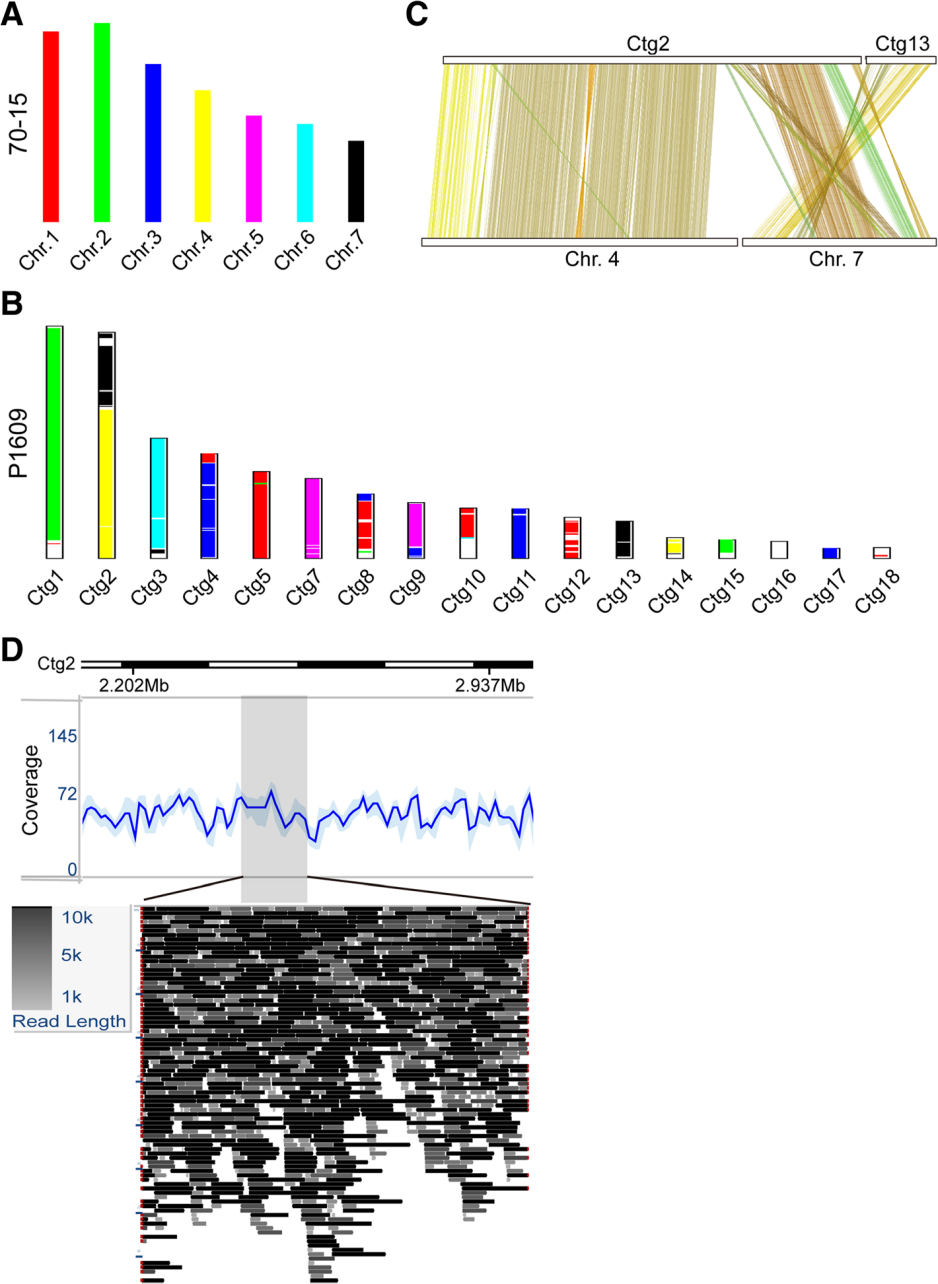
Chromosome rearrangement and splitting between P1609 and 70–15. **a** Bar plot showing the chromosomes in 70–15. **b** Bar plot showing the assembled contigs of P1609. Colinear chromosomes of 70–15 and contigs of P1609 are indicated by the same color. **c** Dual synteny plot showing splitting of Ctg2 of P1609 into chr. 4 and chr. 7 in 70–15. **d** PacBio long-read coverage from 2.02 to 2.94 Mb of Ctg 2. Color of reads indicate different read lengths.

Chromosome rearrangements were broadly documented and had been reported to play important roles in fungal evolution, host adaptation and pathogenicity of phytopathogens [38, 39]. To explore genome collinearity and rearrangement between P1609 and 70–15, the identified collinear gene blocks that linked with different chromosomes in 70–15 (Fig. 3a) were visualized in the contigs (ctgs) of P1609 (Fig. 3b). P1609 and 70–15 overall displayed high genome collinearity. Ctg1, ctg3, ctg5 and ctg7 in P1609 correspond to chr.2, chr.6, chr.1 and chr.5 of 70–15, respectively. We found that the second largest contig in the P1609, ctg2, is a combination of chr.4 and chr.7 of 70–15 (Fig. 3b). The joining region was spanned by multiple single PacBio long reads (Fig. 3d), excluding the possibility that the rearrangement was an artifact due to assembly errors. Meanwhile, notably, the contig end regions of P1609 showed a higher level of chromosome deletion and rearrangement when compared to 70–15 (Fig. 3c). For instance, ctg4 and ctg8 were merged with blocks homologous to 70–15 chr.1 and chr.3 at the end of the contig, while ctg9 was merged with blocks from chr.3 and chr.5 at the end of the contig (Fig. 3b).

## Discussion

Here we sequenced the genome of *P. penniseti* using PacBio SMRT technology. Comparative genomic analysis revealed several chromosome rearrangement events relative to *P. oryzae* and large differences in pathogenicity-related gene repertoires between the *P. penniseti* strain P1609 and the *P. oryzae* strain 70–15.

### Chromosome fission and fusion

Long-read sequencing greatly improved genome assembly and thus provided more valuable details on genome structures at a chromosome level. By comparing the chromosome structure between P1609 and 70–15, we found several chromosome splitting and rearrangement events. Chromosomal rearrangements have been reported to be associated with virulence evolution in several pathogens due to the loss of *AVR* gene(s) [26, 40, 41]. In addition to rearrangements, the telomere regions were also rearranged relative to *P. oryzae*. Further study is required to investigate the role of the chromosome recombination during the adaptation to JUJUNCAO as well as the variation that occurs between individuals in the *P. penneseti* population.

### Unique genes and positively selected genes

In this study, we found 2210 unique genes in *P. penniseti* that were not present in the 70–15 genome. The P1609 genome encodes more CAZymes than 70–15 genome. For example, P1609 contains twice the number of genes encoding glycosyl hydrolase family 28 (GH28) pectinases in 70–15. Generally, necrotrophic plant pathogens possess more GH28 enzymes than biotrophic and non-pathogens fungi [42]. These results suggested that P1609 might heavily rely on CAZymes in the interaction with JUJUNCAO.

To identify genes under positive selection, the Ka/Ks ratios in the 5991 orthologous gene pairs between P1609 and 70–15 were calculated, and only 6 genes with Ka > Ks in P1609 were identified. Functional annotation showed that most of these genes appear to be involved in secondary metabolic pathways. For example, P1609_5032 encodes an isoamyl alcohol oxidase that turns isoamyl alcohol into isovaleraldehyde [43]. In *Saccharomyces cerevisiae*, isoamyl alcohol could induce filament formation [44]. P1609_5032 might play a role in fungal development through controlling the level of isoamyl alcohol. P1609_791 encodes a folylpolyglutamate synthase involved in biosynthesis of folate, required for protein synthesis in bacteria, mitochondria, and chloroplasts, and biosynthetic pathways for purines, dTMP, methionine, and formyl-methionyl-tRNA [45]. P1609_3360 encodes a glycerol uptake protein, which is an important intracellular osmolyte participating in osmotic stress response [46] and critical for the function of appressorium in *P. oryzae* [47]. P1609_7869 encodes a homolog of a spore surface glycoprotein shown to be involved in spore adhesion to hydrophobic surfaces in several *Colletotrichum* species [48–50]. Adhesion of spore tip mucilage is important for infection in *P. oryzae* [51]. P1609_1006 encodes a BclB glycoprotein (collagen-like protein). Its homologs in *Bacillus anthracis* are important components of the infection-associated structure exosporium [52–54], although its role in filamentous pathogens remains unknown. The positive selection on these genes suggested that they might play roles in the interaction between P1609 and JUJUNCAO.

### Putative secreted proteins

Increasingly, studies of pathogen populations support the view that plant immunity is the major force driving the specialization of adapted pathogens [2, 15–18]. It was previously proposed by Schulze-Lefert and Panstruga that effector-triggered immunity (ETI) is the major force driving the host specificity of pathogens [55]. Our previous study focusing on *AVR* gene evolution of the *Pyricularia* species also revealed that directional selection exerted by host plants is the direct force driving host specificity in *Pyricularia* species [18]. Recent studies in the interaction between *M. oryzae* and rice revealed that both ETI and pathogen-associated molecular patterns (PAMPs)-triggered immunity (PTI) are involved in determining effector repertoires and specialization to the two subspecies of *P. oryzae* [15–17]. This opinion was supported by the results that the japonica-isolate genomes contain many effectors to overcome the strong basal immunity posed by japonica rice, but prevent them from infection of indica rice through the triggering of ETI. By contrast, the indica-isolates deserted most of the effectors to avoid recognition by *R* genes encoded by indica plants, but are disabled in conquering the elevated basal immunity in japonica rice [16]. Our comparative genomic analysis showed that P1609 contains a large number of unique effector candidates compared with 70–15, but lost (or never possessed) many putative effectors found in the 70–15 genome, including all known AVR effectors. One possible explanation is that the JUJUNCAO harbors a high level of basal immunity, as well as an arsenal of resistance genes, which drove P1609 to gain host-specific effectors to overcome the robust basal immunity posed by JUJUNCAO. The large difference in effector/Avr gene content between *P. penniseti* and *P. oryzae* suggest the selection driving evolution of these fungi can help direct investigation of the molecular mechanisms underlying the interaction between *Pyricularia* species and their hosts.

## Conclusion

*Pyricularia* species are pathogens of grasses, many of which are either food or forage grasses. The model fungus *P. oryzae* had been well studied. However, there are only a few whole-genome sequences available for other species, such as those from *Pennisetum* grasses. Here, we generated long-read PacBio reads and produced a assemblage with long-continuity contig sequences. The phylogenomic and comparative genomic analysis suggested that P1609 is a *Pyricularia* species genetically distant from *P. oryzae*, and may have employed diverse mechanisms during the adaptation to JUJUNCAO, including the pathways associated with secondary metabolics (positively selected genes), CAZymes and the putative secreted proteins, as well as other PHI proteins. In summary, the P1609 assembly and genome annotation represents the few available *Pyricularia* genome resources for studying the pathogenic mechanism of this fungus towards *Pennisetum* grasses.

## Methods

### Isolation of the fungal strain

The *Pennisetum*-infecting strain P1609 was isolated from the leaf spot lesion of JUJUNCAO (*Pennisetum giganteum* Z. X. Lin), in the nursery of National Engineering Research Center of JUNCAO Technology, Fujian Agriculture and Forestry University located at No. 63 Xiyuangong Road, Minhou County, Fuzhou, Fujian Province, China.

### DNA extraction, amplification and sequencing

To prepare the genomic DNA for sequencing, the P1609 isolate was cultured in the liquid complete medium (CM) in a 110-rpm shaker at 25 °C for 3 to 4 days. The mycelia were then collected for the preparation of genomic DNA using a CTAB method as previously described [18]. Sequencing libraries were prepared using the SMRTbellTM Template Prep Kit 1.0 (PACBIO) and sequenced using PacBio Sequel platform (NovoGene, China).

### Assembly and annotation

De novo sequence assembly was conducted by SMRTLink v. 5.0.1.10424, HGAP 4 pipeline provided by Pacific Bioscience Company. In HGAP 4 pipeline, the expected genome size was set as 45 Mb based on the reported size of *Pyricularia* genomes, and default settings were used for other parameters. Gene prediction was conducted using Fgenesh from SoftBerry (MolQuest II v2.4.5.1135, http://linux1.softberry.com/berry.phtml) with *Pyricularia* additional variants as training organism. Gene functional domain annotation was conducted by InterproScan (version 4.8, http://www.ebi.ac.uk/ interpro/), and PfamScan [56]. Pathogen-Host Interaction (PHI) genes were predicted by performing a whole genome blastp analysis against the PHI database (E < 10^− 10^) [57, 58]. Putative carbohydrate-active enzymes (CAZymes) were identified using the HMMER 3.1b1 by searching annotated HMM profiles of CAZyme families downloaded from the dbCAN database in protein sequences of P1609 [59].

### Repeat analysis

De novo repeat sequence identification was analyzed by using RepeatModeler (version 1.0.8) with default settings. Repeat sequences obtained from RepeatModeler have been used to search for repeat sequences in the P1609 genome by RepeatMasker (version 3.3.0) (http://www.repeatmasker.org/) [60].

### Phylogenetic analysis and comparative genomic analysis

Phylogenomic tree of P1609 and *B. cinerea* [61], *C. gloeosporioides* [62], *F. graminearum* [63], *N. crassa* [64], *P. oryzae* [8], *S. sclerotiorum* [61],*T. reesei* [65] and *U. maydis* [66] was built based on single copy orthologs from clustering result of OrthoFinder (v0.6.1) [27]. 2051 single copy genes have been selected out from 13 organisms in total (see Fig. 2a) and aligned with MAFFT (mafft-linsi-anysymbol) [67]. The phylogenetic tree was constructed using FastTree based on the alignments of single-copy orthologs with approximately-maximum-likelihood model and 100 bootstrap iterations [68]. For divergence time estimation, the phylogenetic analysis was conducted using r8s (version 1.81), and the divergence time of *Pyricularia* and *Neurospora* (200 MYA) was used as a reference [28, 29]. Clustering result of 13 genomes was also used for unique gene identification and comparative genomic analysis. The comparative genomic study of homologs among P1609 and 70–15 (the reference isolate of *P. oryzae*), *N. crassa*, *F. graminearum* and *C. gloeosporioides* was conducted using the OrthoFinder [27]. The identification of positively selected genes was performed as described previously [11]. Genes were aligned in pairs between P1609 and 70–15. Codeml tool in PAML suite was used to calculate Ka/Ks ratio, with the assumption that Ka/Ks ratio > 1 suggested the gene was under positive selection. For the prediction of secreted proteins, SignalP 4.1 was implemented to predict signal peptides and TMHMM 2.0 have been used to predict the transmembrane domain [34, 69]. Proteins with a signal peptide cleavage site, amino acid length smaller than 400 amino acids, and no transmembrane domain after the region signal peptide cleavage site were defined as putative secreted proteins in this study. We used MCScanX to identified syntenic blocks between P1609 and 70–15. To detect the conserved synteny blocks, the reciprocal best-match paralogs of P1609 and 70–15 were conducted by all-against-all BLASTP comparison, with E-value < 10^− 10^ [70].

## Additional files

Additional file 1: **Figure S1.** Gene copy numbers of CAZymes in Botrytis cinereal, Colletotrichum gloeosporioides, Fusarium graminearum, Neurospora crassa, Sclerotinia sclerotiorum, Trichoderma reesei and *Ustilago maydis* as well as Pyricularia isolates collected from *O. sativa* (PoOs), *T. aestivum* (PoTa), *D. sanguinalis* (PgDs), *S. viridis* (PoSv), and *E. indica* (PoEi). Log2 Copy Number presents variation of copy number with increased red color means increased number of CAZymes. (XLSX 20 kb)

Additional file 2: **Table S1.** The CAZymes identified in P1609 and other fungi. (XLS 47 kb)

Additional file 3: **Table S2.** P1609_vs_7015_unique_secreted. (XLSX 16 kb)

Additional file 4: **Table S3.** 70–15 VS P1609 unique secreted proteins. (XLSX 71 kb)

Additional file 5: **Figure S2.** GH28 of P1609 (P1609_11576, P1609_5879, P1609_2497, P1609_5781, P1609_680 and P1609_5514)) and PoOs (MGG_09608, MGG_08752 and MGG_08938), PgDs (Ds0505_9820). Extra copies of GH28 in P1609 is marked by blue. (JPG 237 kb)

Additional file 6: **Table S4.** Predicted PHI in P1609. (JPG 113 kb)

## Abbreviations

*AVR* gene: Avirulence gene
*B. cinerea*: *Botrytis cinerea*
CAZymes: Carbohydrate-active enzymes
CBM: Carbohydrate-binding module family
Cg: *Colletotrichum gloeosporioides* (*C. gloeosporioides*)
chr: Chromosome
CM: Complete medium
CTAB: Cetyltrimethyl ammonium bromide
ctg: Contig
ETI: Effector-triggered immunity
Fg: *Fusarium graminearum* (*F. graminearum*)
GH: Glycosyl hydrolase family pectinases
LTR: Long terminal repeats
MYA: Million years ago
Nc: *Neurospora crassa* (*N. crassa*)
*P. giganteum*: *Pennisetum giganteum*
*P. grisea*: *Pyricularia grisea*
*P. penniseti*: *Pyrucularia penniseti*
*P. penniseticola*: *Pyricularia penniseticola*
*P. setariae*: *Pyricularia setariae*
*P. typhoides*: *Pennisetum typhoides*
PgDs: *Pyricularia* strain isolated from *Digitaria sanguinalis*
PHI: Pathogen-Host Interaction
PL: Polysaccharide Lyase Family
Po: *Pyricularia oryzae* (*P. oryzae*)
PoEi: *Pyricularia* strain isolated from *Eleusine indica*
PoOs: *Pyricularia* strain isolated from *Oryza sativa*
PoSv: *Pyricularia* strain isolated from *Setaria viridis*
PoTa: *Pyricularia* strain isolated from *Triticum aestivum*
PTI: Pathogen-associated molecular pattern (PAMPs)-triggered immunity
*R* genes: Resistance gene
*S. Sclerotiorum*: *Sclerotinia sclerotiorum*
SMRT: Single-Molecule Real-Time
*T. reesei*: *Trichoderma reesei*
TE: Transposable elements
*U. maydis*: *Ustilago maydis*
