# Supplementary figures and images for: Comparative genomic analysis revealed rapid differentiation in the pathogenicity-related gene repertoires between Pyricularia oryzae and Pyricularia penniseti isolated from a Pennisetum grass
Source: BMC Genomics. 2018 Dec 13;19:927. doi: 10.1186/s12864-018-5222-8 (PMC6293661; doi:10.1186/s12864-018-5222-8)

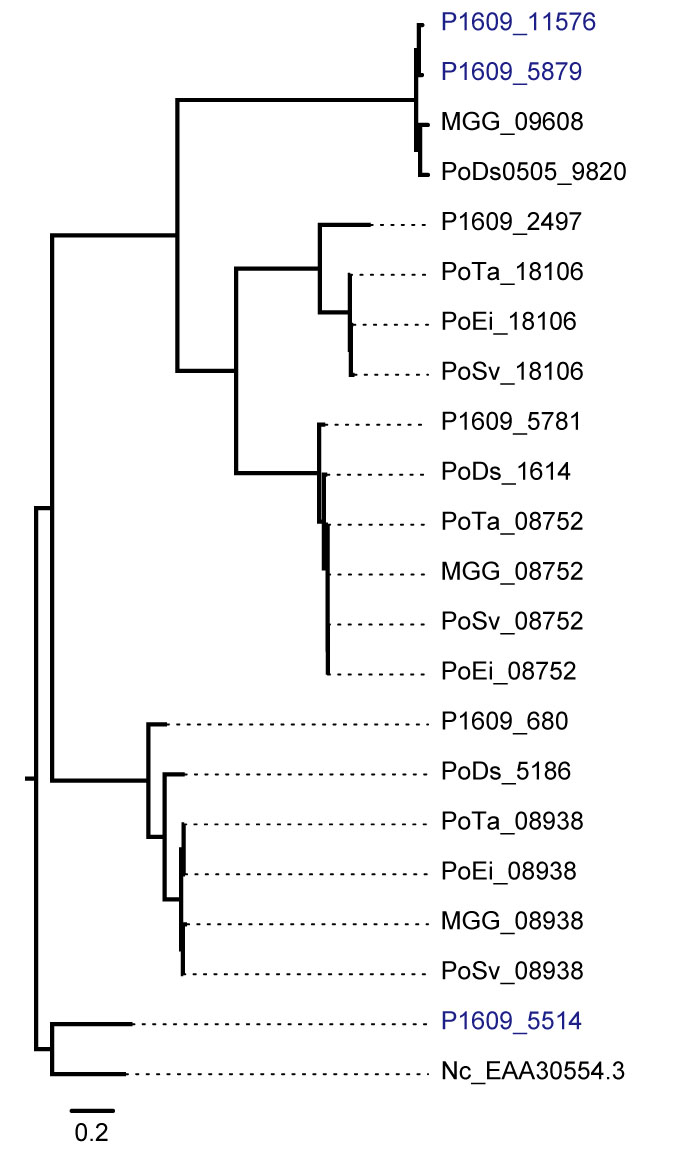

Supplement: Supplementary file 5 — Figure S2. GH28 of P1609 (P1609_11576, P1609_5879, P1609_2497, P1609_5781, P1609_680 and P1609_5514)) and PoOs (MGG_09608, MGG_08752 and MGG_08938), PgDs (Ds0505_9820). Extra copies of GH28 in P1609 is marked by blue. (JPG 237 kb) [file 12864_2018_5222_MOESM5_ESM.jpg]

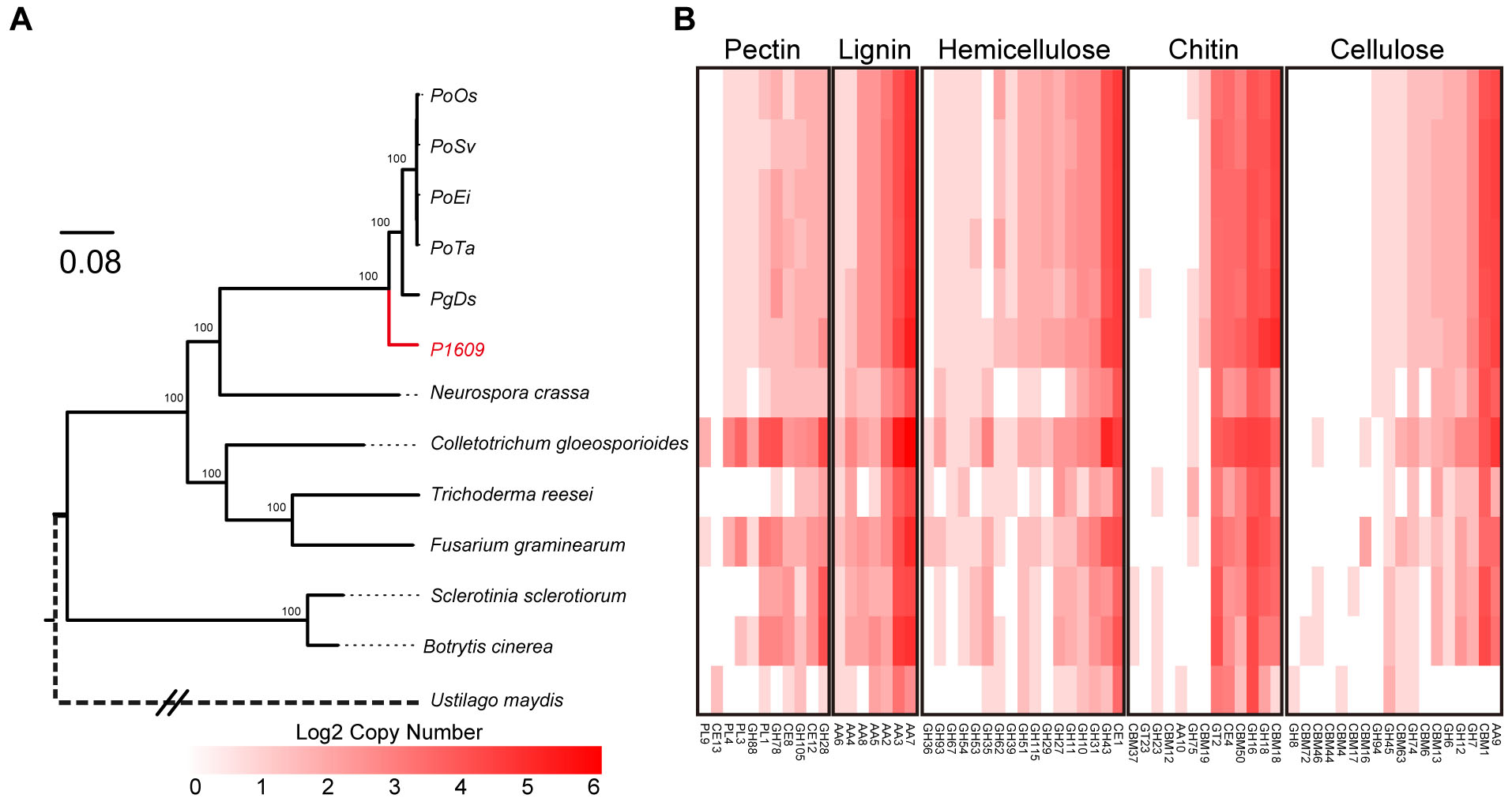

Supplement: Supplementary file 6 — Table S4. Predicted PHI in P1609. (JPG 113 kb) [file 12864_2018_5222_MOESM6_ESM.jpg]
